# Bovine Tuberculosis Epidemiology in Cameroon, Central Africa, Based on the Interferon-Gamma Assay

**DOI:** 10.3389/fvets.2022.877541

**Published:** 2022-07-22

**Authors:** Robert F. Kelly, Lina Gonzaléz Gordon, Nkongho F. Egbe, Emily J. Freeman, Stella Mazeri, Victor N. Ngwa, Vincent Tanya, Melissa Sander, Lucy Ndip, Adrian Muwonge, Kenton L. Morgan, Ian G. Handel, Barend M. D. C. Bronsvoort

**Affiliations:** ^1^Farm Animal Services, The Royal (Dick) School of Veterinary Studies, University of Edinburgh, Edinburgh, United Kingdom; ^2^Epidemiology, Economics and Risk Assessment (EERA) Group, The Roslin Institute, The Royal (Dick) School of Veterinary Studies, University of Edinburgh, Edinburgh, United Kingdom; ^3^School of Life Sciences, University of Lincoln, Lincoln, United Kingdom; ^4^School of Veterinary Medicine and Sciences, University of Ngaoundere, Ngaoundere, Cameroon; ^5^Cameroon Academy of Sciences, Yaoundé, Cameroon; ^6^Tuberculosis Reference Laboratory Bamenda, Hospital Roundabout, Bamenda, Cameroon; ^7^Laboratory of Emerging Infectious Diseases, University of Buea, Buea, Cameroon; ^8^Institute of Ageing and Chronic Disease and School of Veterinary Science, University of Liverpool, Liverpool, United Kingdom

**Keywords:** bovine tuberculosis, *Mycobacterium bovis*, interferon-gamma assay, epidemiology, cattle, Cameroon

## Abstract

Despite sub-Saharan Africa (SSA) accounting for ~20% of the global cattle population, prevalence estimates and related risk factors of bovine tuberculosis (bTB) are still poorly described. The increased sensitivity of the IFN-γ assay and its practical benefits suggest the test could be useful to investigate bTB epidemiology in SSA. This study used a population-based sample to estimate bTB prevalence, identify risk factors and estimate the effective reproductive rate in Cameroonian cattle populations. A cross-sectional study was conducted in the North West Region (NWR) and the Vina Division (VIN) of Cameroon in 2013. A regional stratified sampling frame of pastoral cattle herds produced a sample of 1,448 cattle from 100 herds. In addition, a smaller cross-sectional study sampled 60 dairy cattle from 46 small-holder co-operative dairy farmers in the NWR. Collected blood samples were stimulated with bovine and avian purified protein derivatives, with extracted plasma screened using the IFN-γ enzyme-linked immunosorbent assay (Prionics Bovigam^®^). Design-adjusted population prevalences were estimated, and multivariable mixed-effects logistic regression models using Bayesian inference techniques identified the risk factors for IFN-γ positivity. Using the IFN-γ assay, the prevalence of bTB in the dairy cattle was 21.7% (95% CI: 11.2–32.2). The design-adjusted prevalence of bTB in cattle kept by pastoralists was 11.4% (95% CI: 7.6–17.0) in the NWR and 8.0% (95% CI: 4.7–13.0) in the VIN. A within-herd prevalence estimate for pastoralist cattle also supported that the NWR had higher prevalence herds than the VIN. Additionally, the estimates of the effective reproductive rate *R*_t_ were 1.12 for the NWR and 1.06 for the VIN, suggesting different transmission rates within regional cattle populations in Cameroon. For pastoral cattle, an increased risk of IFN-γ assay positivity was associated with being male (OR = 1.89; 95% CI:1.15–3.09), increasing herd size (OR = 1.02; 95% CI:1.01–1.03), exposure to the bovine leucosis virus (OR = 2.45; 95% CI: 1.19–4.84) and paratuberculosis (OR = 9.01; 95% CI: 4.17–20.08). Decreased odds were associated with contacts at grazing, buffalo (OR = 0.20; 95% CI: 0.03–0.97) and increased contact with other herds [1–5 herds: OR = 0.16 (95% CI: 0.04–0.55); 6+ herds: OR = 0.18 (95% CI: 0.05–0.64)]. Few studies have used the IFN-γ assay to describe bTB epidemiology in SSA. This study highlights the endemic situation of bTB in Cameroon and potential public health risks from dairy herds. Further work is needed to understand the IFN-γ assay performance, particularly in the presence of co-infections, and how this information can be used to develop control strategies in the SSA contexts.

## Introduction

*Mycobacterium bovis* infection, the main cause of bovine tuberculosis (bTB), has been reported in many species, including wild and domesticated animals, but has particular importance in cattle (1, 2). Infection with *M. bovis* does not always result in clinical disease, as many animals live with latent infections without clinical signs (3). Subclinical infection is the most common presentation in endemic settings, which is difficult to detect. Importantly, *M. bovis* is zoonotic, with transmission through living in close contact with cattle or consumption of raw milk or other animal products (4). In humans, *M. bovis* infection is estimated to be responsible for 3.1% of human TB cases globally, often linked with immunosuppressive infections like HIV (5, 6). A small sample of human TB cases screened at a central tuberculosis reference laboratory in the NWR identified three *M. bovis* cases in a sample of 175 human TB cases (1.7%; 95%CI: 0.3–4.9) (*Personal communication*), highlighting the potential transmission from cattle to humans. Many sub-Saharan Africa (SSA) communities have close contact with cattle and consume untreated dairy products putting them at increased risk of *M. bovis* infection. The epidemiology of bTB is poorly described in SSA cattle populations, with limited local or national testing/control programmes in place. However, it is important to have a more granular understanding of bTB epidemiology within specific populations to understand the production impacts and human health risks and to target the regional control efforts (7).

The Central African country of Cameroon is an example of an SSA country where cattle are economically and culturally important for many rural and peri-urban communities (8). Historically, cattle production has been undertaken by the Fulani ethnic group, a pastoral community spanning Central and West Africa (9, 10). Cattle keeping is core to Fulani culture, not only for meat and milk production but importantly as financial capital. The Fulani extensively graze *Bos indicus* cattle breeds and many still adopt nomadic seasonal grazing practices known as transhumance. Meat and milk are sold at cattle markets for local consumption and act as a vital source of protein for urban populations. Over the past 20 years, small-scale dairy farmer co-operatives have appeared, particularly in the Northwest Region (NWR) of Cameroon (11). These dairy farmers tend to be from non-Fulani backgrounds and rear small numbers of *Bos taurus* cattle, mainly Holstein-Frisian type animals, semi-intensively in basic stalled housing. Milk is sold through their farmer co-operative to peri-urban communities at local public markets. Bovine tuberculosis has been described as endemic in the country and poses a significant public health risk due to high levels of milk consumption (11, 12).

Despite the potential zoonotic risk of bTB, in Cameroon, meat inspection for TB lesions is the only means of protecting public health, and there is no routine *ante mortem* diagnostic surveillance in cattle populations. To understand the impacts of bTB within Cameroon, it is important to improve our understanding of the local epidemiology, including the prevalence within specific cattle populations, and to highlight potential risk factors or important co-infections to target control measures where needed. Previous estimates of bTB prevalence, using post-mortem examination (PME) and *ante mortem* diagnostics, vary between 0.1 and 40% (13–19) and risk factors for bTB positivity may vary within cattle populations in Cameroon due to differences in management practices but have been minimally investigated.

Abattoir surveys are essential to highlight the presence of bTB within a population, to gather strains for typing and to assess diagnostic test performance. However, they are inevitably biased and are not representative of the cattle population as a whole, and it can be challenging to collect metadata for risk-factor analyses. Post-mortem detection of lesions is also insensitive and may lead to an underestimation of the prevalence, particularly missing early-stage infections (20). *M. bovis* infections predominatly stimulate cell-mediated immunity (CMI) responses in cattle (21–23); therefore, *ante mortem* immunological diagnostic tests are important tools for bTB epidemiological studies as well as test and cull control programmes. Diagnostic tests used to detect part of this CMI response include the *in-vitro* interferon-gamma (IFN-γ) assay and the *in-vivo* tuberculin skin tests [single intradermal test (SIT) and single comparative intradermal tuberculin test (SCITT)] (20, 24, 25). The SCITT is recognized by the World Organization for Animal Health (OIE) as the primary diagnostic test for *ante mortem* bTB diagnosis (26), with a high specificity (median: 99.5%; CI: 78.8–100%). However, the SCITT has a relatively low sensitivity (median: 50.0%; CI: 26–78%) based on a recent meta-analysis (27), resulting in potentially half the infected animals being missed and strongly suggesting its performance is much lower than previous claims (28, 29). The IFN-γ assay also detects the predominant Th1 immune response to *M. bovis*, and can experimentally detect positive animals 2 weeks post-infection (30). Consequently, the IFN-γ assay is reported to have a higher sensitivity (median: 67%; CrI: 49–82%) than the SCITT with slightly lower specificity (median: 98%; CI: 96–99%) (27). A test with a high sensitivity would be advantageous in estimating the prevalence of a disease that has zoonotic consequences in a setting with no control measures (31–33) to minimize false-negative results. Unlike the skin tests, the IFN-γ assay can be used in the field without the need for a return visit and therefore could be valuable in epidemiological surveys in low- and middle-income settings (LMICs).

In this study, we estimated the prevalence of bTB using the IFN-γ assay for pastoralist and dairy cattle populations. We then identified and quantified potential risk factors for bTB in cattle kept in pastoralist herds, critical to our understanding of bTB epidemiology in Cameroonian cattle populations. Finally, we estimated the effective reproductive number using the age-stratified results for each of the two sites.

## Materials and Methods

### Study Population, Sampling Design, and Methods

#### Pastoralist Livestock Keepers

**Figure 1:**
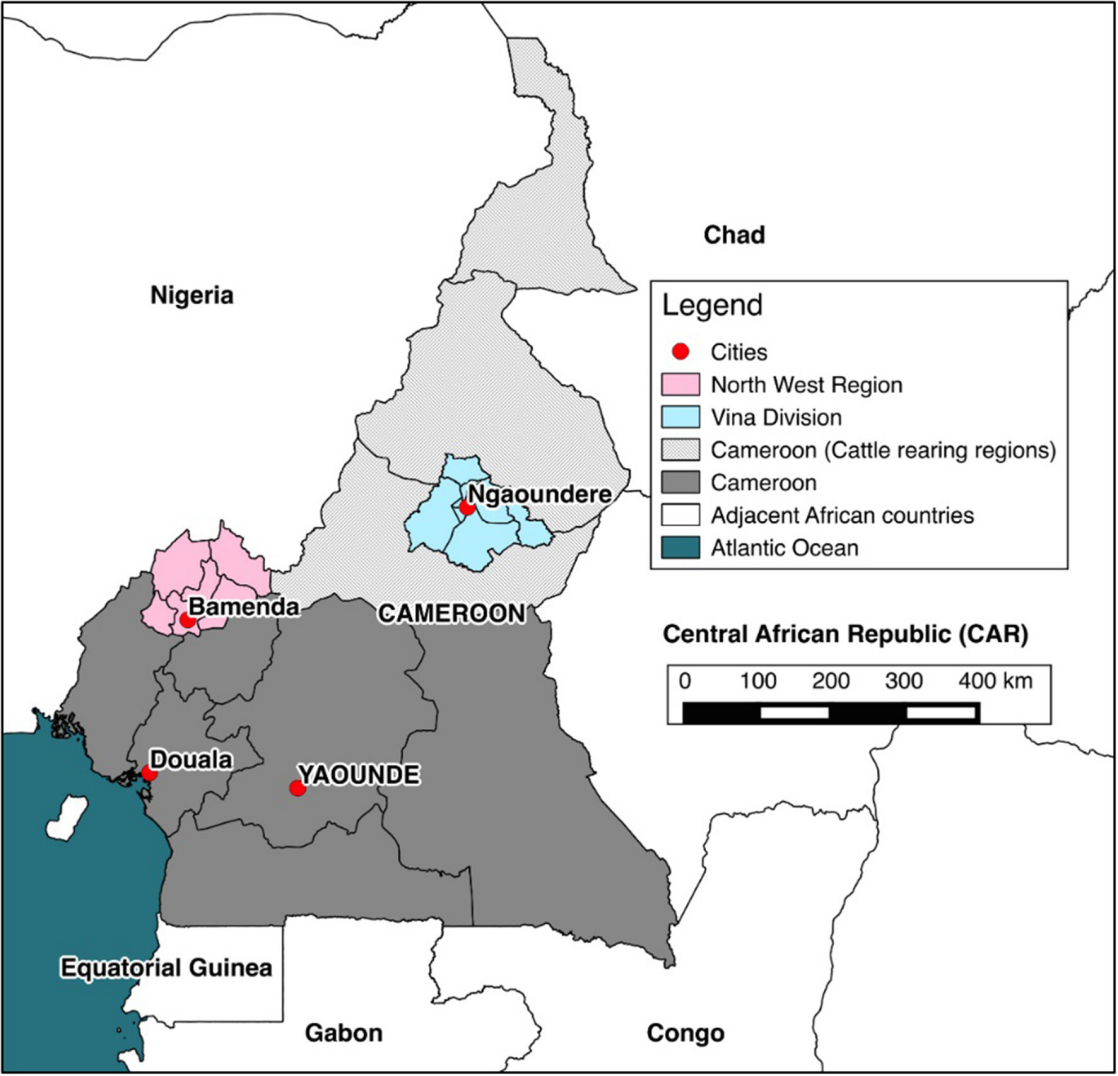
Map of Cameroon. The location of cattle rearing areas (light gray), study sites (pink and blue) and major cities (red).

The study sites were the Northwest (NWR) and Vina Division (VIN) of the Adamawa Region of Cameroon. Both are of similar geographical size of ~17,000 km^2^ (Figure 1) (34). A population-based stratified (by sub-location) random cross-sectional survey was conducted between January–May, 2013 in the NWR and September–November, 2013 in the VIN. The participants were pastoralists whose herds were listed in the Ministry of Livestock, Fisheries and Animal Industries vaccination records at 81 local veterinary centers in the NWR and 31 in the VIN in 2012. A total of 5,053 pastoralist herds in the NWR and 1,927 in the VIN, with a range of 1–215 cattle per herd, were included in the sampling frame. The list of herds in each site was stratified by the administrative area; there are seven divisions in the NWR and eight sub-divisions within the VIN giving roughly equal geographical size for logistical purposes. A random sample of 50 herds was taken from each site and sampling was proportional to the total number of herds listed in each of the divisions/sub-divisions within each of the two sites. The sample size was based on a clustered random sample of cattle assuming a cattle level prevalence of ~10% (35), a within-herd variance of 0.15 and between herd variance of 0.01, an average herd size of 70, a relative sampling cost of 12:1 for herd:cattle and relative error of ±15% (Survey Toolbox; AusVet) (36). This gave a target sample size of 15 cattle per herd and 88 herds, assuming perfect test performance. To allow for potential losses or dropout and to have balanced samples from the two sites, we aimed for 50 herds in each of the two sites in the NWR and VIN. Within each herd, the 15 sampled animals were randomly selected and stratified into three age classes; >6 months to <2 years old (young), 2–5 years old (adult), and older than 5 years (old).

Herds kept by pastoralists were visited either at a site where the cattle grazed or were handled. A local translator explained the project to the pastoralist herdsman or dairy farmer in either Foulfulde, Pidgin English or French. Individuals were asked to give verbal consent to participate in the study in the language in which they were most comfortable. All sampled animals were then examined by the same veterinarian, with data recorded at an individual animal level to accompany samples. These included signalment [sex, age by dentition score (DS), breed and body condition score (BCS)], and if the anthelmintic treatment had been administered in the previous 12 months. The method for aging by DS and BCS was carried out on 5-point scales (35). Dentition score was collapsed into age classifications and animals were categorized as young (<2 years), adult (≥2 and <5 years), and old adult (≥5 years) for the purposes of analysis. “Improved” breed cattle were defined as cattle that had the phenotypic appearance of mixed *Bos indicus* breeding. “Exotic” cattle defined as those cattle that had the phenotypic appearance or which were reported by the farmer to be fully or mixed *Bos taurus*. Plain and heparinised blood samples were collected from the jugular or tail vein of the selected animals. Then, a herd-level questionnaire was administered through an interview in the respondent's preferred language, to collect data on husbandry practices relating to the herd, dairying practices, knowledge and awareness of infectious diseases. The total number of cattle present and location using the GPS coordinates (using Garmin eTrex^®^ Venture) were also recorded.

#### Smallholder Dairies in the North West Region

The second survey was of the small-scale dairy farmers who were all registered with the Ministry of Livestock, Fisheries and Animal Industries. The address list for 2012 was obtained from their NWR office in Bamenda. Dairy co-operatives were established as part of a non-governmental organization (NGO) initiative in the 1990s to improve milk production in the area. Donated Holstein-Friesian cattle from Ireland and Kenya were imported and given to families to be reared in zero-grazing systems (37). Calves born from these original cattle were then passed on to other members joining the co-operatives. There were 229 dairy farmers grouped into 13 co-operatives with 3–52 farmers per co-operative. The co-operatives were categorized geographically into four groups, with three being sampled as they represented separate co-operatives. The fourth group consisted of four widely dispersed co-operatives and was not sampled for logistical reasons. Thus, 164 dairy farmers were included in the sample frame. A stratified (by co-operative group) random sample of dairy farmers was selected proportional to the number of dairy farmers in each group. Based on the assumption of perfect test performance, a prevalence of ~6% in adult cattle (35) and each dairy farmer having two adult cows resulting in a sample size of 46 dairy farmers (Survey Toolbox; AusVet) (36). Dairy herds were visited at the homestead, sampled in the same way as pastoral cattle.

### Interferon-γ Assay and Diagnostic Tests for Other Infectious Diseases

The IFN-γ assay (31) was carried out following the manufacturer's instructions as follows. Three aliquots of 1.5 ml heparinised blood per animal were incubated with either 15 μl of avian purified protein derivative (PPD), bovine PPD (Prionics^®^ Lelystad Tuberculin PPD) or PBS for 24 h at 37°C within 8 h of sampling. Cultured blood was then centrifuged at 300 *g* for 10 min, plasma aliquotted and subsequently stored at −20°C in a portable travel freezer. Electrical supplies were maintained by electricity, portable generators or vehicle batteries, wherever required in the field. Plasma samples were transported at −20°C to the Laboratory of Emerging Infectious Diseases (LEID), University of Buea, Buea, Cameroon to conduct the IFN-gamma ELISA. Reagents were reconstituted where appropriate and samples were allowed to reach room temperature (22 ± 5°C). The avian PPD, bovine PPD, and PBS previously stimulated plasma samples were diluted 1:1 with dilution buffer. Diluted plasma samples were added to the pre-coated 96-well plate along with duplicates of positive, negative and PBS control kits. The ELISA procedure followed the manufacturer's instructions (38). By standard interpretation, as per commercial kit instructions, animals with a bovine PPD plasma sample of ≥0.1 than avian PPD and PBS indicate the presence of *M. bovis* infection.

As part of the risk factor analysis, we were interested in the associations with co-infections, especially infections that may be immunosuppressive [bovine viral diarrhea (BVD), bovine enzootic leukosis (BLV)] or could potentially influence the interpretation of the IFN-γ assay [*M. avium subspecies paratuberculosis* (paraTB)]. After collection, all serum samples were heat-treated at 56°C for 120 min and stored at −20°C until tested. Serum samples were then tested for exposure to various infections using serological ELISAs. Screening for antibodies to BVD Virus (BVDV) was conducted using the ID Screen^®^ BVDV Serum Competitive ELISA (39) following the manufacturer's instructions. The competition percentage (S/N%) was calculated as the OD for the sample divided by the OD for the negative control times 100 to convert to a percentage. The manufacturers suggest samples S/N% ≤ 40% are considered positive; S/N% >40 and ≤ 50% are considered inconclusive and S/N% >50% are considered negative. For this study, samples S/N% ≤ 40% were considered positive and S/N% >40% were considered negative. Screening for Bovine Leukemia Virus (BLV) exposure was conducted using the ID Screen^®^ BLV Serum Competitive ELISA (40) following the manufacturer's instructions. The competition percentage (S/N%) was calculated as for the BVDV ELISA. The manufacturers suggest samples S/N% ≤ 50% are considered positive; S/N% >50 and <60% are considered inconclusive and S/N% ≥60% are considered negative. For this study, samples with an S/N% ≤ 50% were considered positive and those with an S/N% >50% were considered negative. Screening for antibodies to MAP was conducted using the ID Screen^®^ Paratuberculosis Serum Indirect Multi-species ELISA (41) following the manufacturer's instructions. The sample to positive ratio (S/P%) was calculated by (ODsample—ODNC)/(ODPC—ODNC) ×100. The manufacturers suggest samples S/P% ≤ 60% are considered negative; S/P% >60 and <70% are considered inconclusive and S/P% ≥70% are considered positive. For this study, samples S/P% <70% were considered negative and S/P% ≥70% were considered positive.

### Statistical Analysis

All information was initially recorded onto paper forms, which were later transferred into a relational Access database (Microsoft Access^®^). The herd-level descriptive results have been published previously (34). All statistical analyses were performed using packages and functions in R version 3.6.1 (42). Graphics were produced using the *ggplot2* package (43). Spatial data were displayed using the QGIS 2.2^®^ (44) or *tidyverse* collection of R packages (45) and shapefiles obtained from the GADM database of Global Administrative Areas (www.gadm.org). The structure of the pastoralist survey was incorporated into analyses using the *svydesign, confint*, and *svyby* functions in the *survey* package (46). This allowed the stratified study design to be accounted for in the prevalence estimates. Confidence intervals (CI) and Bayesian credibility intervals (CrI) are reported at a 95% level throughout and statistical significance was defined at *P*-values ≤ 0.05. Throughout the paper, we refer to the apparent prevalence as the “prevalence” for ease of reading. The “true” prevalence after adjusting for the test imperfections can be crudely approximated by multiplying the reported prevalence estimates by 1.5.

The effective reproductive number (*R*_t_*)* was estimated as follows. The average life expectancy was estimated as $\frac{1}{\mu }=\overset{\infty }{\underset{x=1}{\sum }}l_{x}$, where *l*_*x*_ is the survival rate at age *x* and is calculated as the ratio of the number of animals at age *x* divided by the number of animals in age class 1 (animals aged up to 1 year old). For an individual of age *a*, the standard SIR (susceptible, infectious and recovered) model predicts that the probability that an individual is still susceptible is given by S(*a*) = exp[–*a*μ(*R*_t_-1)], where *a* is the animal's age and μ is 1 over the average life expectancy. As the numbers of the susceptible and infectious are binomially distributed, the likelihood function of these numbers was obtained as a function of *R*_t_. *R*_t_ was then inferred as the value that maximized the logarithm of this likelihood function:


$$\begin{array}{l} logL\left(R_{t}\right)=\sum {}_{i=0}^{n}\log\left(\exp\left(-a_{i}\mu \left(R_{t}-1\right)\right)\right)\\ \;+\sum {}_{i=0}^{m}\log(1-\exp\left(-b_{i}\mu \left(R_{t}-1\right)\right)) \end{array}$$

where *n* is the number of susceptible animals (sero-negative) of age *a*_1_, …, *a*_*n*_, and *m* is the number of sero-positive animals (ages *b*_1_, …, *b*_*m*_) for all animals (47). Due to the homogeneity of the dairy cattle sample, this analysis was only conducted for pastoralist cattle.

Risk factors for IFN-γ assay positivity were investigated in pastoralist cattle using multivariable mixed-effects logistic regression models run using a Bayesian framework. The outcome was the individual animal bTB IFN-γ status. A backward stepwise model-building approach was used for model specification (48). Explanatory variables of interest collected using the herdsman questionnaire were identified *a priori* by the authors and included host factors, husbandry-related features and risk contacts based on their presumed influence on the risk of exposure to *M. bovis*. Specifically, among the variables of interest were contacts with wildlife, such as warthogs, antelope and buffalo, going on the seasonal transhumance, the size of the herd and the number of other herds contacted at grazing. IFN-γ status with exposure to co-infections was also explored, including BVD, BLV and paraTB. Age, sex and site (NWR or VIN) were included as potential confounders. Non-informative prior distributions for the parameters were used as recommended by Gelman et al. (49), including the intercept and the random effect (herd). The data and the priors were combined to estimate a posterior distribution using Markov Chain Monte Carlo (MCMC) scheme. The Watanabe-Akaike Information Criteria (WAIC) and Leave-One-Out CrossValidation Criteria (LOO-IC) were used to compare the models and select the optimal model (the model that better describes the outcome balancing parsimony and fit). Model convergence and stability checks were performed through the inspection of trace and autocorrelation plots of the MCMC samples for each chain. Graphical analysis of binned residuals was used to perform a predictive posterior check (50). Finally, a receiver operating curve (ROC) was used to test the ability of the model to discriminate between IFN-γ positive and negative animals. This was built using the *pROC* package (51). The Bayesian models were implemented using functions included in the *brms* package in R; this package uses “Stan” for full Bayesian inference (52). Posterior distributions were estimated for each explanatory variable and their effects are summarized using the mean and 95% Credible Intervals (CrI). The exponential transformation was used to generate the odds ratio (OR) for epidemiological interpretation.

### Ethical Statement

The study was reviewed and approved by the University of Edinburgh Ethics Committee, UK (ERC No: OS02-13) and by the Institute of Research and Development (IRAD), Cameroon. All participants gave informed verbal consent to the translator before participating and could opt-out at any stage.

## Results

**Figure 2:**
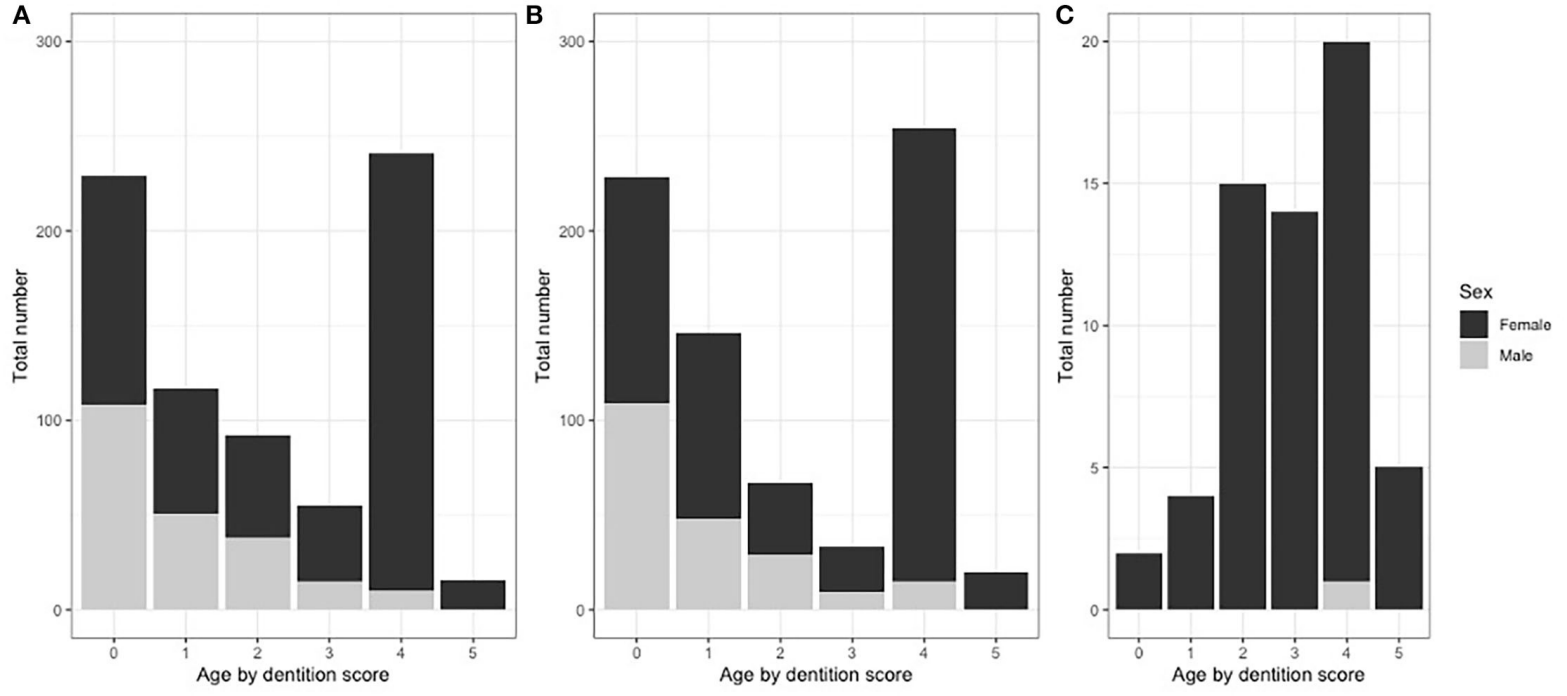
Proportions of cattle sample by dentition score, sex and study site grouping. **(A)** NWR pastoral cattle (*n* = 750), **(B)** VIN pastoral cattle (*n* = 748), and **(C)** NWR dairy cattle (*n* = 60).

In total, 100 pastoralist herds were recruited: 50 in the NWR and 50 in the VIN. Of these 100, 23 were replacements from the same veterinary center for herds that declined or were unable to participate in the study. All 46 selected dairy farmers participated and none were replaced. In total, 750 pastoralist kept cattle were sampled from 50 herds (15 per herd) in the NWR and 748 from 50 herds (14–15 per herd) in the VIN. In the dairy cross-sectional study, 60 cattle (1–4 per herd) were sampled from 46 dairy farmers. For pastoralist cattle, there were equal numbers of males and females in the young age group and mainly females in older age groups, as expected with the slaughter of males from 3 to 4 years of age for consumption (Figure 2). The dairy herds were very small, and all animals in a herd were sampled. A detailed summary of animal- (53) and herd-level data (34) have been previously published.

### Prevalence of IFN-γ Positive Pastoralist and Dairy Cattle

**Figure 3:**
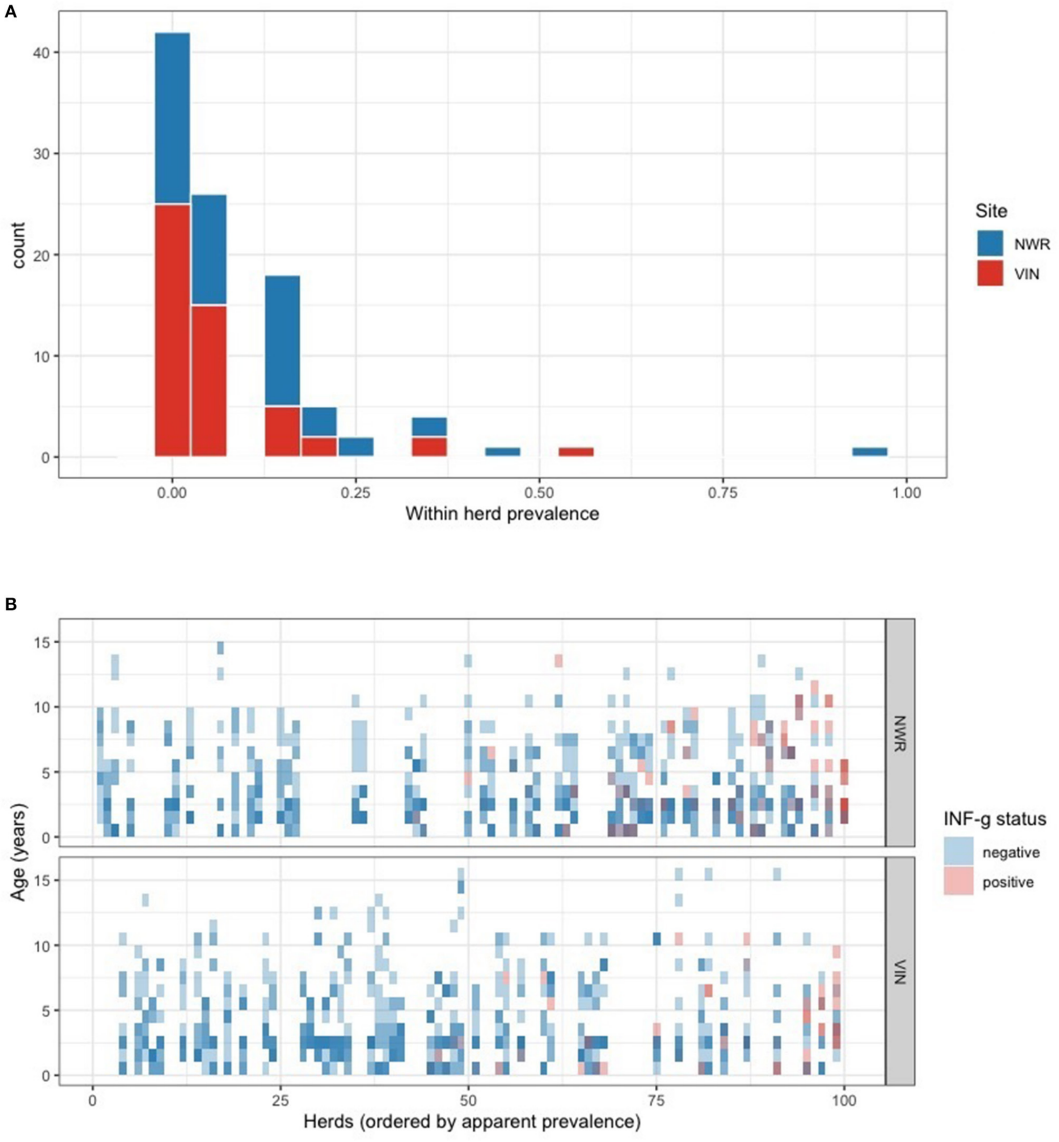
**(A)** Within-herd IFN-γ positivity prevalence distribution stratified by the two pastoralist sites in the NWR and VIN. **(B)** Tile plot showing each individual animals IFN-γ positivity status (blue = negative, red = positive). Animals are grouped together in vertical columns according to their herd and the herds are ordered from right to left from highest to lowest within-herd prevalence.

The overall animal-level true prevalences were 11.4% (95% CI: 7.6–17.0) in the NWR, 8.0% (95% CI: 4.7–13.0) in the VIN, and the apparent prevalence in dairy animals in the NWR was 21.7% (95% CI: 11.2–32.2). Due to the homogeneity of the dairy cattle sample, the remainder of the analysis focused on pastoralist cattle (NWR and VIN). A within-herd prevalence estimate generated from the raw proportions and the distribution across the NWR and VIN pastoralist herds are presented in Figure 3A. The individual animal status grouped by herd and ordered by within-herd prevalence is presented in Figure 3B. The trend demonstrates that the NWR appears to have higher prevalence herds than the VIN, which is consistent with the overall prevalences.

**Figure 4:**
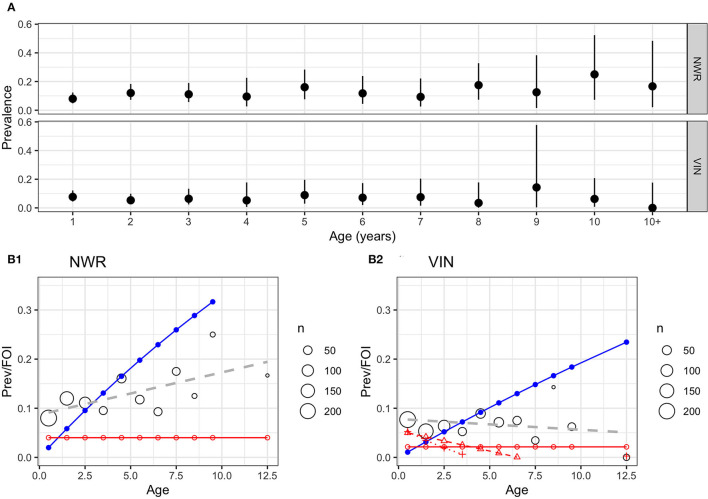
**(A)** Age-stratified IFN-γ positivity prevalence by age (years) with unadjusted 95% CI for the two pastoralist cattle keeping sites in NWR and VIN. **(B1,B2)** Age-stratified prevalence of IFN-γ positivity prevalence in cattle in two sites in Cameroon in 2013. The predicted prevalence based on a simple linear age function (blue line) and a constant force of infection (λ) (red line) based on the Muench model. The black circles show the mean prevalence for that age strata with the size proportional to the number of animals in that age strata and a simple linear regression model of IFN-γ positivity with age in years (gray dashed line).

The age-stratified prevalences are presented in Figures 4A,B. There appears to be little increase in prevalence with age in the VIN, while there is a slight increase with age in the NWR with the slope of the prevalence (linear regression coefficient = 0.009) statistically >zero (*P* = 0.03) based on a simple linear model of prevalence by age. This lack of increase with age may suggest a relatively low force of infection (FOI) meaning most cattle acquire infection in their first year of life. The FOIs (blue lines Figure 4B) are not consistent with a constant FOI and this supports the hypothesis that most transmission occur when the animals are very young. The estimates of the effective reproductive rate *R*_t_ were 1.12 for the NWR and 1.06 for the VIN, adding to the evidence that there are potentially different endemic patterns in the different sites and that there is a higher transmission rate in the NWR.

**Figure 5:**
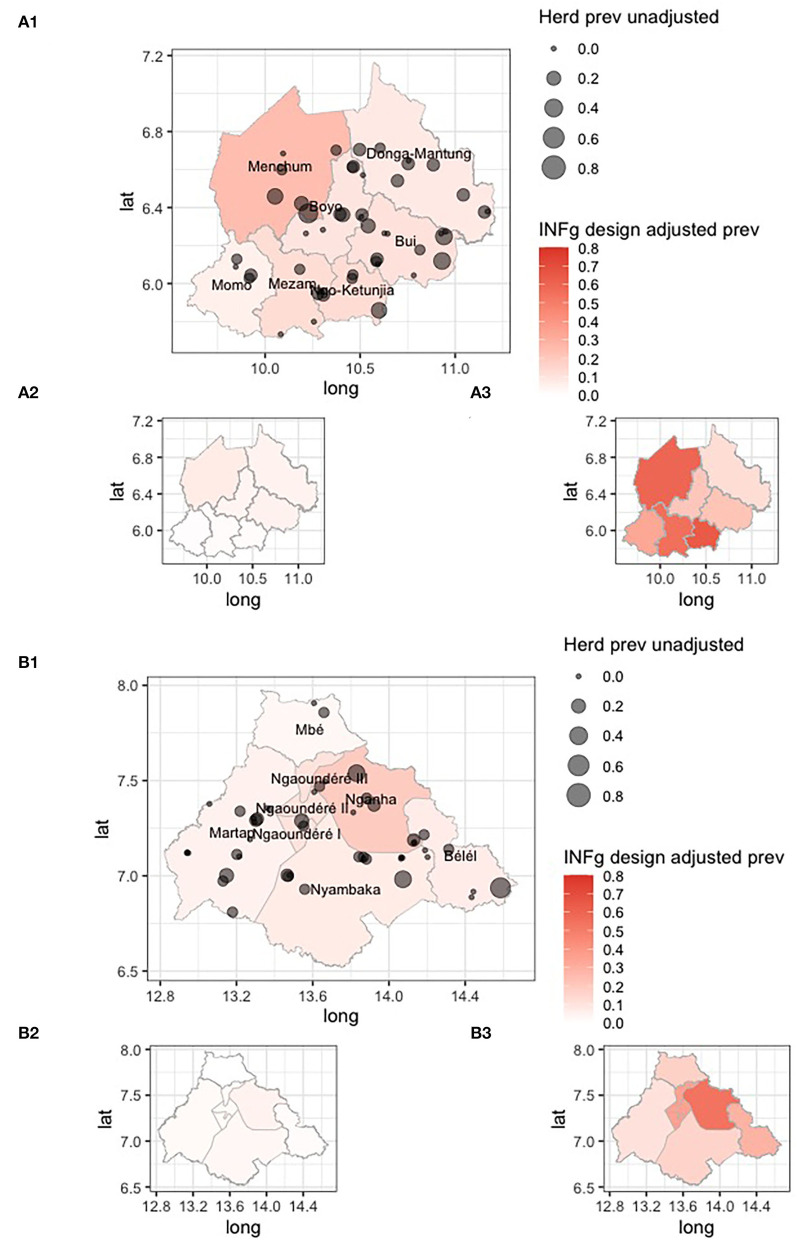
Choropleth maps of the Northwest Region (NWR) **(A1)** and Vina Division (VIN) **(B1)** in Cameroon colored by design adjusted IFN-γ prevalence for the administrative strata, overlaid with the approximate location of individual herds sized by the raw proportion of animals positive within each herd. The smaller inset choropleth maps are for the lower **(A2,B2)** and upper **(A3,B3)** 95% confidence intervals, respectively.

The design-adjusted prevalences by division/sub-division are given in Table 1 and mapped in Figure 5. They show a wide range of IFN-γ prevalences, with a very low prevalence in Mbé in the north of the VIN and a high prevalence in Menchum in the northwest of the NWR.

**Table 1:** Design-based animal-level seroprevalence (not adjusted for test performance) of RVF antibodies in cattle in two sites in Cameroon in 2013 stratified by Division (NWR) and sub-Division (VIN).

**Division/sub-division**	**Raw proportions (positive/sample)**	**Design-based seroprevalence (%)**	**Design-based 95% CI (%)**
**Northwest region (NWR)**
Boyo	8/90	9.6	4.0–21.0
Bui	18/195	10.9	5.1–21.7
Donga-Mantung	14/180	7.8	4.8–12.4
Menchum	15/75	24.6	6.8–59.2
Mezam	19/105	12.6	1.6–55.3
Momo	4/60	5.4	0.7–33.1
Ngoketunjia	6/45	13.1	1.3–64.2
**Vina division (VIN)**
Belel	12/150	6.7	1.3–28.5
Martap	11/255	5.0	2.2–11.0
Mbé	1/30	2.7	0.0–17.3
Ngan-Ha	8/73	19.2	4.7–53.4
Ngaoundere	5/60	10.1	2.3–35.2
Nyambaka	12/180	6.9	2.8–15.7

### Risk Factors for bTB in Pastoralist Cattle in Cameroon Based on the IFN-γ Assay

**Figure 6:**
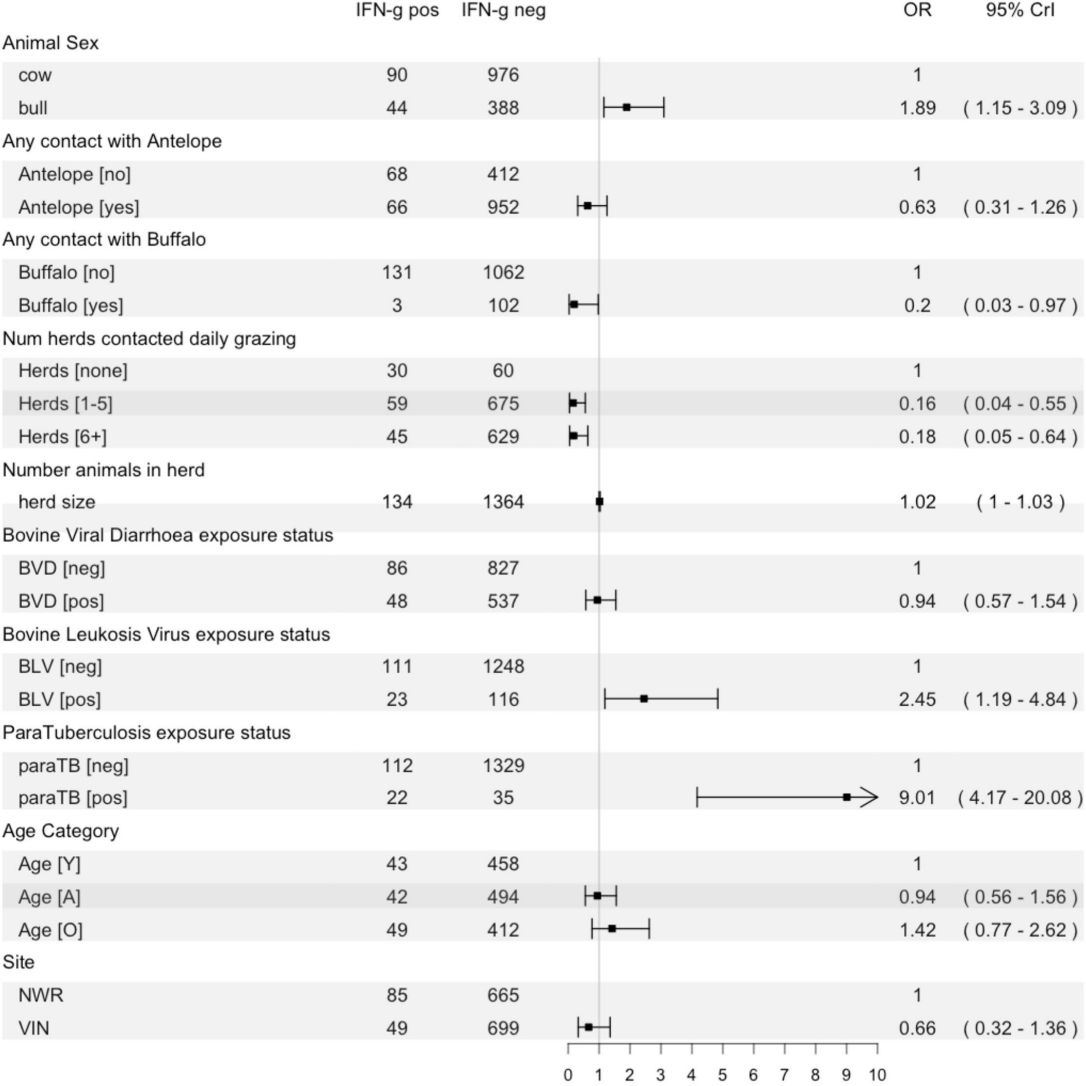
Forest plot showing the fixed effects in the final multilevel model of risk factors for IFN-γ positivity with the raw counts for each variable, a plot of the odds ratio with 95% CrI and the numeric result and CI.

A multivariable logistic regression model was developed to explore host, husbandry and contact risk factors for IFN-γ positivity (Figure 6). The final model suggests that bulls have almost twice the odds of being IFN-γ positive compared to cows (OR = 1.89; 95% CI:1.15–3.09). Contact with buffalo was associated with a reduced risk (OR = 0.20; 95% CI: 0.03–0.97), and contact with antelope was statistically significant (OR = 0.63; 95% CI: 0.31–1.26). The odds of positivity increased with herd size (OR = 1.02 for each additional herd contacted on an average day; 95% CI:1.01–1.03), and there was an unexpected protective association between the number of herds contacted at grazing and positivity [1–5 herds: OR = 0.16 (95% CI: 0.04–0.55); 6+ herds: OR = 0.18 (95% CI: 0.05–0.64)]. Finally, there was no evidence of an important association with BVD exposure, but there was increased odds of positivity if an animal was also seropositive to BLV (OR = 2.45; 95% CI: 1.19–4.84) or paraTB (OR = 9.01; 95% CI: 4.17–20.08). The area under the ROC curve for the final averaged models was 0.86 (95% CI: 0.82–0.89), indicating good model performance in relation to classifying IFN-γ assay positive cattle using this dataset.

## Discussion

This study revealed there was a high prevalence of bTB based on the IFN-γ assay, which is comparable to the previously reported estimates in other regions of SSA (54–57). This was the first time the test was used in Cameroon. There was also marked variation across the administrative regions. The assay has the advantage of requiring only a single visit to an animal that has obvious logistical advantages in these remote settings. Laboratory facilities to incubate and harvest the plasma within <8 h should not be underestimated when using the diagnostic in remote field settings.

The high level of IFN-γ assay positivity is likely to reflect a high prevalence of *M. bovis* infection across all cattle populations where there is a lack of control measures. We previously highlighted concerning levels of lesions detected at slaughter in our associated abattoir-based studies in NWR and VIN (35). Interestingly, the lesion-based studies in the abattoir showed a higher prevalence of bovine TB lesions at the Ngaoundere abattoir in the VIN (11.33%) compared to that in the Bamenda abattoir in the NWR (3.99%). The finding here shows the reverse, a lower positivity on the IFN-γ assay in the VIN. This is likely due to the difference in the age structure of cattle taken to the abattoir as discussed in our earlier work, i.e., at the Ngaoundere abattoir majority of the slaughtered animals were older female animals. Consequently, differences in prevalence estimates between abattoir studies and stratified cross-sectional studies could be influenced by differences in slaughter practices between the two study sites and catchment area of abattoirs being beyond the identified study area. Earlier reports using the SCITT (13) also showed a higher animal-level prevalence in the NWR (5.58%, 95% CI: 3.89–5.44%) compared to the VIN (2.57%, 95% CI: 1.42–3.72) (58), which is consistent with the current observations. Indeed, related cross-sectional studies show a similar relationship, with prevalence in the NWR (20.95%) being greater than in the VIN (0.55%), although this difference seems extreme compared to the other studies (53–56). Some of the differences with the current study can be explained by the study design, diagnostic test or cut-off value differenced; therefore, a direct comparison should be made with prudence and care. For example, differences in prevalence estimates between abattoir studies and stratified cross-sectional studies could be influenced by differences in slaughter practices between the two study sites and catchment area of abattoirs being beyond the identified study area.

This is the first report of bTB in the dairy cattle population from the NWR, and it indicates a potential public health problem of *M. bovis* transmission in urban populations. Particularly considering the relatively poor understanding of bTB and limited pasteurization practices in this group of livestock keepers may facilitate transmission to urban human popualtions where demand for dairy products is on the rise (34).

A key question is whether bTB has reached some sort of endemic stability or is in an epidemic phase in Cameroon. It is probably important also to view the country as a complex network of transmission, driven by local on cattle movements. In this regard, one would expect that bTB in cattle populations from NWR should be stable, given the geographical isolation from other areas of cattle trade (8). On the other hand, a highly dynamic disease pattern in VIN given the continuous cattle movement due to transhumance from the Central African Republic, Chad toward the western Sahel (59). Previous studies have analyzed the market network in Cameroon, and it was very consistent with this notion (8, 59). The molecular analysis also revealed a high *M. bovis* strain diversity mostly due to singleton strains in the VIN with limited evidence of cattle population overlap between VIN and NWR (60). The conclusion from our previous work was that lower rates of transmission were driving the locally expanding infections of bovine tuberculosis in NWR and was characterized by a lower strain diversity dominanted by SB0953 spoligitype (60). The local expansion of bovine tuberculosis in NWR could be due to a high density of cattle population in this isolated region, which increases the opportunity of contact between cattle likely infected with SB0953 spoligotype. Unlike other studies (61–63), there is no increased infection rate with age, which could be because our study did not have the statistical power to detect this if it exists, or indeed this could represent an early-in life exposure to the disease. The VIN exhibited the opposite, a lower ERN, a higher diversity, and a lower recent transmission index, which likely represents a stable endemic status of bovine tuberculosis.

The use of IFN-γ assay highlights significant infection of Cameroonian cattle with *M. bovis* and provides an opportunity to investigate potential risk factors associated with infection. This is particularly interesting because IFN-γ assay can detect *M. bovis* infection weeks or months earlier than the SCITT test (23, 64). The analysis showed that female cattle had lower odds of being identified as IFN-γ positive. This observation is in contrasts with findings from the previous abattoir study, where female cattle were twice as likely to be lesion positive (35). This could be because female cattle are kept longer for reproductive purposes, which means they can be chronically infected, i.e., with gross lesions visible at abattoir inspection but anergic to the IFN-γ assay immunologically. It is also very clear there was a marked difference in the age structure of the populations observed passing through the abattoir compared to at pasture.

Contact with wildlife was associated with lower odds of being IFN-γ positive, i.e., pastoralist cattle grazing with antelope and buffalo was protective, so such cattle were less likely to test positive of the IFN-γ. Although susceptibility of Cameroonian antelope species has not been specifically investigated, antelope species have been reported infected with *M. bovis* (65) like buffalo in South Africa (66), and in some cases implicated as part of the transmission cycle in parts of SSA (67). It is not clear why, therefore, contact with wildlife should be protective but could be related to cattle being grazed in remote areas where there is generally increased space and reduced stocking density. Buffalo are only found in the remote areas of Cameroon, and herds visiting these areas are at much lower densities potentially, explaining this negative association (author observation). Statistically, the associations are non-significant for antelope and, although significant for buffalo, the number of cattle in contact were extremely small, which may reflect a false association. However, risk of other zoonotic bacterial infections has been positively associated with wildlife contact, such as *Brucella species* (53, 68). Consequently, it is difficult to interpret the real importance of wildlife contact and *M. bovis* transmission, with future studies needing to focus on the multiple species to understand transmission dynamics.

Similarly, the association between increasing numbers of contacts at grazing and decreased odds of positivity is difficult to interpret as it seems counter-intuitive. Extensive management practices may reduce the transmission pressure of *M. bovis* transmission compared to intensive cattle rearing systems (69). As most cattle in this study are grazed on communal pastures (34), contamination from *M. bovis* may be diluted by large grazing areas (56) and sunlight desiccation of *M. bovis* (70). It is possible that the increasing number of contacts at grazing may be a proxy for environmental factors or extensive management of pastoral cattle that may reduce *M. bovis* transmission not captured by the questionnaire. Supported by the result that a larger herd size was associated with increased odds of positivity, as would be expected in most infectious diseases (71) usually related to increased infection pressure if an infected animal is present. Future surveillance studies involving cattle, other livestock and free-roaming animals are required to explore the potential dynamics of infection between domestic, wildlife species and their environment.

A particularly interesting result is the association between BLV and paraTB exposure status/seropositivity and IFN-γ/bTB infection status. Given the cross-sectional nature of this study, we were not able to determine the sequence of exposures to know if, for example, BLV preceded exposure to bTB and this induced immunosuppression making the animal more susceptible. There is not much mentioned in the literature about co-infection with BLV (28), hence it is difficult to interpret the full impact of this result. More is understood about the association of paraTB [caused by *Mycobacterium avium subspecies parastuberculosis* (MAP)] and bTB, for example, it has been observed that in populations with higher rates of paraTB or related non-tuberculous mycobacteria (NTM), the IFN-γ test specificity poorly lead to a large number of false positives (72, 73). The very strong association observed here may be more to do with the impact of MAP or NTM co-infection on test performance than the actual risk of infection or disease. Overall, these results highlight that without controlling for exposure to other co-infections, when interpreting the IFN-gamma assay results, are likely to hinder the progress of future bTB surveillance in Cameroon.

Using IFN-γ assay in a representatively sampled naturally infected cattle population, we were able to explore the prevalence and factors which influence bTB positivity in Cameroonian pastoralist cattle populations. We were unable to do this for smallholder dairy cattle due to the relatively small sample and homogeneity of this cattle population. Overall, there is a need for the assessment of IFN-γ assay diagnostic test performance in all Cameroonian cattle populations using Bayesian non-gold standard methods to improve the accuracy of prevalence estimates and risk factor identification. As a follow-up to this work, we are developing a mathematical model integrating molecular and immunological parameters to further unravel the dynamics of bovine tuberculosis in Cameroon.

## Conclusion

Bovine tuberculosis is endemic in pastoralist and dairy cattle populations in Cameroon. Its transmission may be related to a wide range of differences in cattle-keeping practices within the country. Here, we present evidence supporting our previous work in Cameroon on the difference in epidemiological dynamics at play in the two sites. The current study suggests that these two settings are associated with high and low IFN-γ assay positivity rates, respectively. This assay could be of value in LMIC settings where the need for a single visit only has substantial logistical and financial implications. However, further work is needed to understand the associations observed between co-infections with BLV and paraTB and the potential impact on test performance that might need to be accounted for in interpreting the use of the IFN-γ assay in these populations.

## Data Availability Statement

The raw data supporting the conclusions of this article will be made available by the authors, without undue reservation.

## Ethics Statement

The studies involving human participants and animal study was reviewed and approved by the University of Edinburgh Ethics Committee, UK (ERC No: OS02-13) and by the Institute of Research and Development (IRAD), Cameroon. All participants gave informed verbal consent to the translator before participating and could opt out at any stage. Written informed consent for participation was not required for this study in accordance with the national legislation and the institutional requirements. Written informed consent for participation was not obtained from the owners because many of the participants were illiterate so verbal consent was granted and recorded on paper questionnaires.

## Author Contributions

BB, LN, and VT conceived the original project. RK, KM, LN, IH, VT, MS, and NE designed the field study, the databases, and the survey instrument. RK, NE, VN, VT, KM, and BB developed the field SOPs and collected the data. RK, EF, and LG conducted laboratory work. RK and BB cleaned the initial dataset. RK, LG, BB, IH, and SM contributed to the analysis. RK was responsible for writing the initial drafts. All authors contributed comments for the final draft.

## Funding

This work was funded by the Wellcome Trust (WT094945).

## Conflict of Interest

The authors declare that the research was conducted in the absence of any commercial or financial relationships that could be construed as a potential conflict of interest.

## Publisher's Note

All claims expressed in this article are solely those of the authors and do not necessarily represent those of their affiliated organizations, or those of the publisher, the editors and the reviewers. Any product that may be evaluated in this article, or claim that may be made by its manufacturer, is not guaranteed or endorsed by the publisher.
